# Label-Free Infrared Spectral Histology of Skin Tissue Part I: Impact of Lumican on Extracellular Matrix Integrity

**DOI:** 10.3389/fcell.2020.00320

**Published:** 2020-05-12

**Authors:** Lise Nannan, Valérie Untereiner, Isabelle Proult, Camille Boulagnon-Rombi, Charlie Colin-Pierre, Ganesh D. Sockalingum, Stéphane Brézillon

**Affiliations:** ^1^Université de Reims Champagne-Ardenne, Laboratoire de Biochimie Médicale et Biologie Moléculaire, Reims, France; ^2^CNRS UMR 7369, Matrice Extracellulaire et Dynamique Cellulaire, Reims, France; ^3^Université de Reims Champagne-Ardenne, Cellular and Tissue Imaging Platform, Reims, France; ^4^Laboratoire de Pathologie, Centre Hospitalier Universitaire de Reims, Reims, France; ^5^BASF Beauty Care Solutions France SAS, Pulnoy, France; ^6^Université de Reims Champagne-Ardenne, BioSpecT-BioSpectroscopie Translationnelle, EA7506, UFR de Pharmacie, Reims, France

**Keywords:** lumican, extracellular matrix, skin remodeling, infrared imaging, histology

## Abstract

Proteoglycans (PG) play an important role in maintaining the extracellular matrix (ECM) integrity. Lumican, a small leucine rich PG, is one such actor capable of regulating such properties. In this study, the integrity of the dermis of lumican-deleted *Lum*^–/–^ vs. wild-type mice was investigated by conventional histology and by infrared spectral histology (IRSH). Infrared spectroscopy is a non-invasive, rapid, label-free and sensitive technique that allows to probe molecular vibrations of biomolecules present in a tissue. Our IRSH results obtained on control (WT, *n* = 3) and *Lum*^–/–^ (*n* = 3) mice showed that different histological structures were identified by using K-means clustering and validated by hematoxylin eosin saffron (HES) staining. Furthermore, an important increase of the dermis thickness was observed in *Lum*^–/–^ compared to WT mice. In terms of structural information, analysis of the spectral images also revealed an intra-group homogeneity and inter-group heterogeneity. In addition, type I collagen contribution was evaluated by HES and picrosirius red staining as well as with IRSH. Both techniques showed a strong remodeling of the ECM in *Lum*^–/–^ mice due to the looseness of collagen fibers in the increased dermis space. These results confirmed the impact of lumican on the ECM integrity. The loss of collagen fibers organization due to the absence of lumican can potentially increase the accessibility of anti-cancer drugs to the tumor. These results are qualitatively interesting and would need further structural characterization of type I collagen fibers in terms of size, organization, and orientation.

## Introduction

Skin is the largest organ of the human body. It constitutes a physiological and physical barrier and has different functions of protection, thermoregulation, sensitization, excretion, and absorption. However, it is prone to numerous pathologies such as cutaneous skin cancers including melanoma. It is mainly composed of three layers with the epidermis, the dermis, and the hypodermis (Dréno, 2009). The epidermis is a squamous keratinized stratified epithelium. It constitutes the superficial layer of the skin and has a protective function. It is separated from the dermis by a basal lamina: the dermal–epidermal junction itself is involved in the wound healing process. The dermis is a fibrous connective tissue, elastic and highly vascularized. It consists of papillary dermis, reticular dermis and appendages of the skin such as sweat glands and sebaceous glands as well as hair follicles. The papillary dermis is superficial and loose and consists of fine collagen fibers intertwined perpendicular to the epidermis. It also contains elastic fibers. Small blood vessels from the vascular plexus constitute a place of nutritive exchange with the deep layers of the epidermis. The papillary dermis is located above the reticular dermis. The latter is deep and consists of fibers intertwined collagen, in large irregular bundles, horizontally (Ueda et al., 2019). It also contains thick elastin fibers, blood vessels, nerves as well as nerve endings. The hypodermis constitutes the last and deepest layer of the skin. It is composed of subcutaneous white adipose tissue, an energy pool based on a muscular layer. Fibroblasts are the place of synthesis of macromolecules of the extracellular matrix (ECM) including proteoglycans (PGs; Postlethwaite and Kang, 1998). Extracellular matrix of the dermis is mainly constituted of type I and III collagens and PGs.

Lumican belongs to the family of small leucine-rich proteoglycans (SLRP). In the skin, it is a glycoprotein of 57 kDa but in the cornea, it is a PG with glycosaminoglycan (GAG) chains of keratan sulfate. Other major members of the SLRP family are decorin, biglycan and fibromodulin. The papillary dermis is rich in fibroblasts, the principal place of synthesis of SLRP including lumican. The latter is associated with collagen fibrils and especially with type I collagen. Homozygous mice deleted for Lumican gene were generated by Chakravarti et al. (1995, 1998). These mice exhibit a particular phenotype characterized by a fragile and elastic skin and an opacified cornea. This particular phenotype is explained by the abnormal organization of collagen fibers. They have larger diameters and interfibrillar space, demonstrating the architectural role of lumican in maintaining ECM integrity. Together, PGs and lumican regulate the assembly of collagen fibrils in many tissues. In humans, these phenotypic characteristics are comparable to those observed in the case Ehlers-Danlos syndrome. In addition, lumican has been shown to exhibit anti-tumoral activity in melanoma (Brézillon et al., 2007). Furthermore, the involvement of α2β1 integrin has been demonstrated in the modulation of lumican related cell invasion (D’Onofrio et al., 2008; Zeltz et al., 2010). Thus, during the adhesion of the tumor cells to a coating of lumican, a reorganization of the cytoskeleton is observed (Radwanska et al., 2008).

Classically the architectural modification of the dermis matrix is evaluated by conventional histology, biochemical analysis, and immunohistochemistry (Chakravarti et al., 1995, 1998). In this study, we present a novel approach based on infrared spectral imaging (IRSI) to assess morphological and chemical changes in the dermis. Infrared spectroscopy is a vibrational spectroscopy method that is used to characterize simple samples in different forms (solid, liquid, or gas) but also more complex systems such as cells or tissues based on the principle of interaction between light and matter. It is non-invasive and does not require any special preparation. Moreover, it is rapid, label-free, easy to use, and very sensitive (Mainreck et al., 2012). Spectral signatures are related to vibrations of molecular bonds that allow to characterize and determine molecular structure and composition. Coupled with a microscope, it gives maps/spectral images that enable to associate each pixel element to an entire IR spectrum. Infrared spectral imaging (IRSI) is a fast-growing technique and is intensively developed today for cell (spectral cytology) and tissue (spectral histology, IRSH) characterization (Draux et al., 2009; Baker et al., 2014). Spectral information can be used to identify specific biomarkers for disease diagnosis. For example, it has been used for differentiating between spinocellular cancer (SCC) and basocellular cancer (BCC) skin cancers and for demarcating the peritumoral area of melanoma (Ly et al., 2008). It has been successful in discriminating between healthy and cancerous tissues in colon cancer (Nallala et al., 2012).

In this report, the role of lumican on the organization the dermis matrix was undertaken. In parallel to the conventional histology, infrared spectral imaging was applied on skin tissue sections from control wild type (WT) Lum^+/+^ and Lumican-deleted Lum^–/–^ mice to better characterize the dermis remodeling.

## Materials and Methods

### Animal Care

*Lum*^–/–^ mouse line was generated by targeted mutation and fixed to the C57BL/6J genetic background (B6.129S-Lumtm1Chak/J; Chakravarti et al., 1995). This study was performed in compliance with “The French Animal Welfare Act” and following “The French Board for Animal Experiments.” Experiments were conducted under approval of the French “Ministère de l’Enseignement Supérieur et de la Recherche” (ethics committee n°C2EA-56) in compliance with the “Directive 2010/63/UE.”

### Preparation of Tissue Samples

**FIGURE 1:**
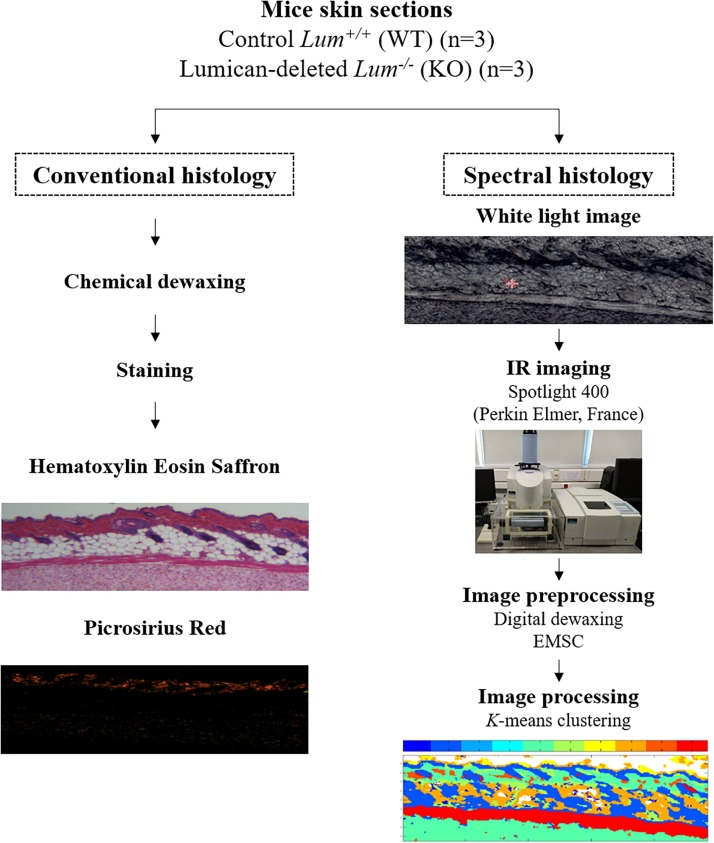
Workflow of conventional and label-free infrared spectral histology of skin tissue.

Skin samples were taken from the ventral flanks of C57BL/6J mice aged 2 to 3 months of control *Lum*^+/+^ (WT) and homozygous mice deleted for Lumican gene *Lum*^–/–^ (KO) with 3 mice per group, respectively. Skin samples were fixed in 4% formalin solution and paraffin embedded (FFPE). Three serial sections of 5 μm thick were cut from the blocks for conventional histology [Hematoxylin eosin saffron (HES)], picrosirius red (PSR), and IRSH. The first section was stained by HES to observe skin histology. Eosin being acidic stains the cytoplasm in pink while the basic hematoxylin stains the nucleus in purple. Saffron dye makes it possible to differentiate between connective tissue and muscle which is not the case for HE. The second section was placed on a 1 mm thick calcium fluoride (CaF_2_) window for IRSI analysis. It can be noted that the IRSI method does not require any staining as structural features are revealed by the intrinsic biomolecular information. The third one was stained with PSR to observe type I collagen fibers (see workflow in Figure 1). Picrosirius red is one of the best understood histochemical techniques able to selectively highlight collagen networks. The collagen fibers are specifically appraised with polarized light detection. Indeed, the special dye PSR has the ability to enhance the natural birefringence of the collagen when exposed to polarized light.

### Microscopic Observations

HES stained sections were imaged with the Olympus IX70 microscope with a 5x objective. Picrosirius red stained sections were imaged with a Zeiss Axiovert 200 M microscope equipped with a 4x objective in polarized illumination to highlight type I collagen fibers. All microscopy images were digitized using a iScan Coreo (Roche Ventana, Meilhan, France).

### IR Spectral Imaging of Mice Skin Sections

All tissue sections *Lum*^+/+^ and *Lum*^–/–^ were imaged in transmission mode using the Spotlight 400 infrared imaging system at a spatial resolution of 6.25 μm/pixel (see workflow in Figure 1). The acquisition parameters were: spectral range from 4000 to 750 cm^–1^, spectral resolution of 4 cm^–1^ and 8 scans/pixel. Calcium fluoride was used because it is transparent in the mid-infrared. The region of interest of the sample was first chosen using the white light image from the infrared microscope and then the IRSI acquisition was started. Prior to this, a background spectrum was measured in a blank area of the CaF_2_ which was then automatically subtracted from each pixel spectrum of the sample.

### IR Image Preprocessing

Since FFPE tissues were used and no chemical deparaffinisation was performed, a mathematical approach of digital dewaxing developed in-house in Matlab software (The Mathworks, Natick, MA; Ly et al., 2008) was carried out as paraffin gives strong FTIR peaks (Ly et al., 2008). Prior to this, all IR images underwent an atmospheric correction using the Spectrum Image 6.4 software (Perkin-Elmer). This step reduces the absorption of molecules present in the sample environment such as carbon dioxide or water vapor. The digital dewaxing procedure included the acquisition of a paraffin image under the same conditions as the sample. This image was used as a target in the Extended Multiplicative Signal Correction (EMSC) algorithm digital dewaxing model. It was used to estimate the contribution of the paraffin in each pixel of the image and therefore only the tissue variability was considered in the processing step such as clustering analysis. The EMSC algorithm also included correction of the baseline and variations related to the difference in sample thickness. Indeed, this step allowed the removal of spectral and spatial artifacts that can influence the spectral image analysis. All spectral images were preprocessed and processed in the reduced spectral region from 1800 to 800 cm^–1^.

### IR Image Processing

Spectral image analysis was based on the *K*-means clustering algorithm. This unsupervised and non-hierarchical technique allows to group pixel spectra into distinct classes (clusters) based on the spectral distance (similarity). Hence, similar data will be grouped in a same cluster and a pixel can be attributed to only one cluster. Each pixel is found associated with a class; a *K*-class. The number of classes is chosen by the user. Each class has a centroid which is chosen randomly at the beginning of the process. Each pixel spectrum is compared to the centroids and regrouped according to the closest similarity. This procedure is iterated until all the pixel spectra are attributed to a given class and until all centroids reached convergence, i.e., are stabilized. Finally, each class is represented by a color and the cluster image is reconstituted as a false color map. In order to compare different skin section images from *Lum*^+/+^ and *Lum*^–/–^ a common *K*-means was employed using 5 and 10 classes.

### Correlation of IR Spectral Images With Type I Collagen Spectrum

The correlation of skin section spectral images with type I collagen spectrum was performed using the Spectrum Image 6.4 software (Perkin-Elmer). To do so, a spectrum of type I collagen was recorded from a 5 μm thick section of FFPE rat-tail tendon and correlated pixel by pixel with an atmospheric-corrected image of skin tissue section. The result was given as a correlated image with a correlation scale ranging from 0 (dark color) to 1 (white color).

### Statistical Analysis

Statistical analyses were performed using Student’s *t*-test and the results were expressed as the mean ± SEM using Prism 8.2.1 (GraphPad Software, La Jolla, CA, United States). The results were considered statistically significant when *^∗∗^p* < 0.01.

## Results

### Comparison Between Conventional and Label-Free Infrared Spectral Histology in Skin Tissue From Mice Control *Lum*^+/+^ (WT) vs. Lumican-Deleted *Lum*^–/–^ (KO)

**FIGURE 2:**
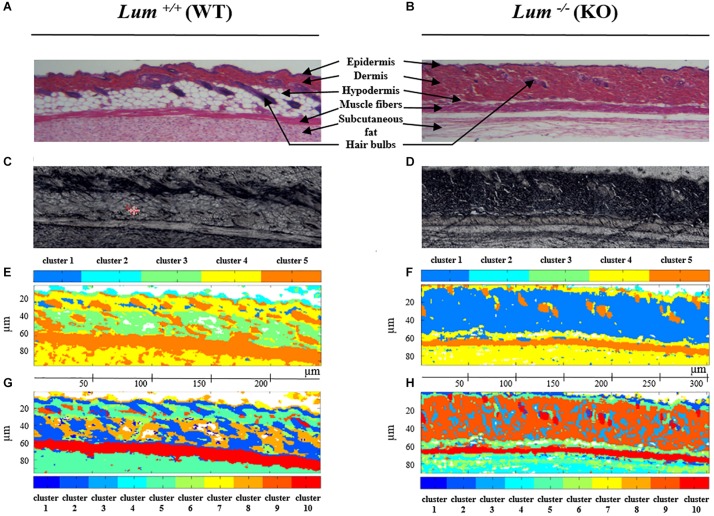
Comparison between conventional and label-free infrared spectral histology of skin tissue from control *Lum*^+/+^ and Lumican-deleted *Lum*^–/–^ mice. **(A,B)** HES staining of skin sections. **(C,D)** Corresponding white light images on CaF_2_ window. **(E,F)** Representative color-coded *K*-means clustering images with 5 classes using the entire mid-infrared spectral range (1800–800 cm^–1^). **(G,H)** Representative color-coded *K*-means clustering images with 10 classes using the entire mid-infrared spectral range (1800–800 cm^–1^).

The HES stained sections (Figures 2A,B) highlight the different skin structures. The epidermis is stained in purple and characterized by a thin outer lining. The dermis appears in pink below the epidermis and is characterized by the presence of hair bulbs. The papillary dermis and the reticular dermis are not distinguishable at low magnification. The hypodermis (in white) is highlighted by the presence of adipose tissue. Underneath the hypodermis, the dark pink layer is composed of muscle fibers recognizable by their elongated shape. The subcutaneous fat is present under the muscle fiber layer. By comparing Figures 2A,B, it can be noticed that *Lum*^+/+^ skin section appears more compact and its dermis is about 2 times thinner compared to *Lum*^–/–^ mice. In addition, the layer of adipose tissue is reduced resulting in a thinner hypodermis in *Lum*^–/–^ compared to *Lum*^+/+^ skin tissue sections. This histological comparison is highlighted with a higher magnification in Supplementary Figure 1. The HES stained sections (Figures 2A,B) and the white light images (Figures 2C,D) are used as reference images for comparison with IRSH obtained by the common *K*-means classification with 5 (Figures 2E,F) and 10 classes (Figures 2G,H). The spectral images equally show different histological structures of the skin such as the epidermis, dermis, hypodermis, muscle fibers, subcutaneous fat and hair bulbs (see legend Figures 2A,B). It is possible to distinguish these different structures with the pseudo-colors obtained by the common *K*-means classification in the spectral images. In the case of the clustering with k = 5 classes, the dermis in *Lum*^+/+^ skin tissue sections (Figure 2E), is mainly represented by cluster 4 (yellow) while in *Lum*^–/–^ skin tissue sections (Figure 2F), it is mainly represented by cluster 1 (dark blue). In both cases, the hair bulbs are identified by cluster 5 (orange). In the case of the clustering with *k* = 10 classes, the dermis is mainly represented by cluster 5 (blue green) in *Lum*^+/+^ skin tissue sections (Figure 2G) and by cluster 9 (dark orange) in *Lum*^–/–^ mice (Figure 2H). The common *K*-means clustering results obtained with 10 classes show a higher heterogeneity in the whole tissue section while keeping the correspondence with conventional histology.

### Reproducible Remodeling of the Dermis Architecture Revealed by Infrared Imaging in Different Groups *Lum*^+/+^ (WT) *vs Lum*^–/–^ (KO)

**FIGURE 3:**
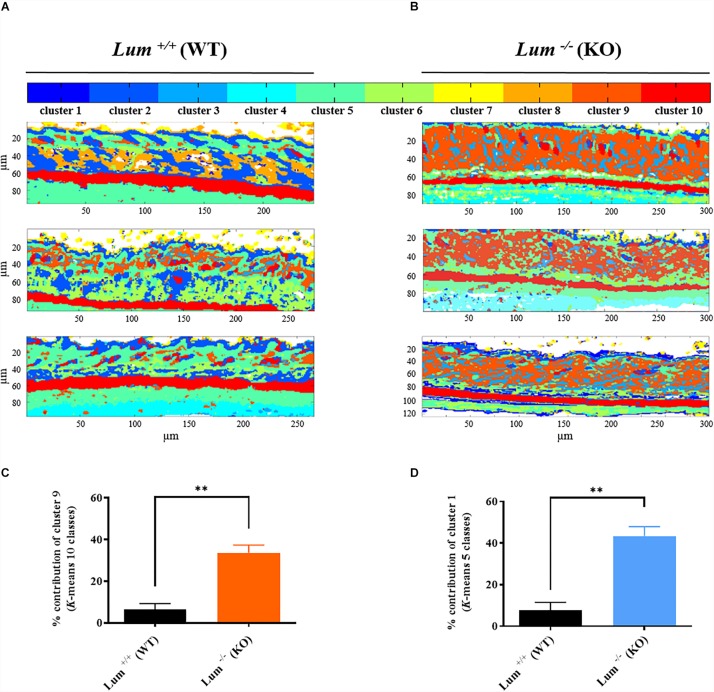
Infrared spectral imaging reveals dermis remodeling from different groups control *Lum*^+/+^ and Lumican-deleted *Lum*^–/–^ mice. **(A,B)** Representative color-coded *K*-means clustering images with 10 classes using the entire mid-infrared spectral range (1800–800 cm^–1^). **(C)** Histogram showing results from statistical analysis of relative contribution of cluster 9 after *K*-means clustering with 10 classes (mean ± SEM, *t* test, **p < 0.01). **(D)** Histogram showing results from statistical analysis of relative contribution of cluster 1 after *K*-means clustering with 5 classes (mean ± SEM, *t* test, **p < 0.01).

In order to verify the above hypothesis, the measurements were repeated on three independent mice skin sections in each group using the common *K*-means clustering with 10 classes. As shown in Figure 3A, all three *Lum*^+/+^ skin sections exhibit a thin dermis and similar spectral images with a homogeneity of pseudo-colors and tissue structures. In contrast, all three *Lum*^–/–^ skin sections (Figure 3B) are characterized by a thicker dermis and exhibit similar pseudo-colors within this group. In addition, the comparison of the two mice groups, shows a loss of the integrity of the skin dermis in the *Lum*^–/–^ group and an intergroup heterogeneity suggesting a remodeling of the dermis architecture in the *Lum*^–/–^ group.

These qualitative observations enable to determine two specific clusters in relation with the dermis structure: clusters 1 and 9 for K-means with 5 and 10 classes, respectively. Differences in the percentage distribution of clusters between the two groups of mice are represented in the form of a histogram. The percentage of cluster 9 (in orange) corresponding to dermis is the only one to increase significantly by 5.3-fold (^∗∗^*p* < 0.01) in the *Lum*^–/–^ group compared with *Lum*^+/+^ control group (Figure 3C and Supplementary Table 1). Similar results have been obtained by common *K*-means with 5 classes (Supplementary Figure 2) where an intragroup homogeneity is observed. The intergroup comparison reveals for cluster 1 (blue) a significant 5.6-fold increase (^∗∗^*p* < 0.01) in the *Lum*^–/–^ group compared to *Lum*^+/+^ control group (Figure 3D and Supplementary Table 2). However, the classification with 10 classes appears better as it improves the differentiation of histological structures and reveals more molecular signatures that were represented by only one cluster in the clustering with 5 classes. In this classification, cluster 9 is associated with the dermis and the observed increase correlates with that obtained for cluster 1 using a *K*-means classification with 5 classes.

### Infrared Spectral Correlation of Type I Collagen With Skin Tissue Remodeling

**FIGURE 4:**
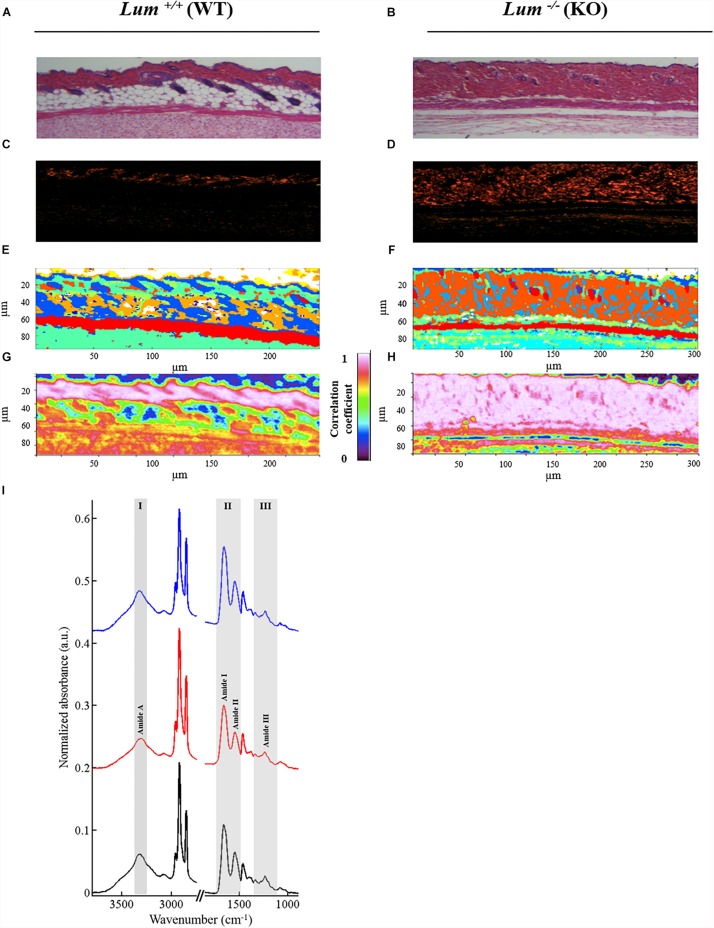
Correlation of the type I collagen spectral signature with skin dermis by infrared imaging. **(A,B)** HES staining and **(C,D)** picrosirius red staining of skin sections (objective 63x). **(E,F)** Representative color-coded *K*-means clustering images with 10 classes using the entire mid-infrared spectral range (1800–800 cm^–1^). **(G,H)** IR correlation maps using reference spectrum from type I collagen of rat tail tendon. **(I)** Comparison between type I collagen reference spectrum (black line) with spectrum taken randomly from the dermis of (*Lum*^+/+^) (red line) and (*Lum*^–/–^) (blue line) mice skin tissues.

In order to compare the contribution of type I collagen in the skin tissue of both mice groups (*Lum*^+/+^ and *Lum*^–/–^), we performed HES staining (Figures 4A,B), picrosirius red staining (Figures 4C,D) and IRSH (Figures 4E,F). HES staining confirmed the higher dermis thickness in *Lum*^–/–^ compared to *Lum*^+/+^, which is explained by a disorganization of type I collagen fibers as revealed by picrosirius red staining. Comparison of HES, picrosirius red stainings with the IRSH strongly suggests that cluster 9 (in orange) mainly corresponds to type I collagen fibers (stained in red). In order to better observe the contribution of type I collagen in spectral images, a correlation image (Figures 4G,H) was computed with a representative spectrum of type I collagen obtained from a rat-tail tendon included in paraffin.

Figure 4I shows the comparison of a representative spectrum of type I collagen obtained from a paraffin-embedded rat-tail tendon with a representative spectrum taken from the dermis of *Lum*^+/+^ and *Lum*^–/–^ mice. Three characteristic spectral zones of collagen bands are highlighted in gray between 3600–3200 cm^–1^ (zone I: amide A), 1700–1500 cm^–1^ (zone II: amides I and II), and 1330–1204 cm^–1^ (zone III: amide III). The latter is composed of a triplet 1330, 1280, and 1204 cm^–1^. The other peaks are assigned to paraffin. These spectra allow identifying by IRSH the presence of collagen in the dermis. The correlation images are shown in Figures 4G,H with a color scale which varies from 0 (low correlation) to 1 (high correlation). A strong correlation coefficient of 0.973 and 0.980 is observed in the dermis respectively for the *Lum*^+/+^ and *Lum*^–/–^ mice groups. These correlation images demonstrate a strong contribution of type I collagen in the dermis in both groups of mice with however a thicker dermis in *Lum*^–/–^ compared to *Lum*^+/+^ mice as previously described. These results corroborate with those obtained by staining with picrosirius red.

## Discussion

This work reports on the role of lumican on the organization of the dermis matrix in WT Lum^+/+^ and Lumican-deleted Lum^–/–^ mice. Lumican plays an important role in maintaining the ECM integrity (Chakravarti et al., 1995). Different approaches combining conventional histology and IRSH have been undertaken to characterize the skin tissue remodeling. In order to compare spectral images with conventional histology, it is necessary to apply multivariate data analysis, here for instance *K*-means clustering, to extract morphological and chemical features. *K*-means clustering is an unsupervised method that minimizes intra-cluster variation and allows to compare several images together to show inter- and intra-group structural modifications (Sebiskveradze et al., 2018). In a first approach comparing HES staining and IRSH of the skin of mice *Lum*^+/+^ and *Lum*^–/–^ shows a good correspondence of different histological skin structures from the epidermis to the subcutaneous fat. An important increase in the dermis is observed in *Lum*^–/–^ mice. Chakravarti and collaborators showed in the same skin model structural differences and suggests a disorganized and loose dermis in Lumican-deleted mice (Chakravarti et al., 1995). Similarly, in other organs like the heart, lumican was shown to be important for survival, cardiac remodeling and fibrosis in response to pressure-overload (Mohammadzadeh et al., 2019, 2020). Our results demonstrate that IRSH can identify such structural changes in a label-free manner. Interestingly, these observations were reproducible in the dermis of all *Lum*^–/–^ mice. Furthermore, IRSI of *Lum*^+/+^ and *Lum*^–/–^ mice skin sections obtained after common *K*-means classification with 5 and 10 classes, allowed to highlight specific clusters of the dermis able to discriminate WT and Lumican-deleted mice (clusters 1 and 9, respectively).

Finally, the contribution of collagen type I was evaluated by HES and picrosirius red staining and correlating each pixel of the spectral images with a representative spectrum of type I collagen. The overall results show a very strong correlation of type I collagen in the dermis by both conventional histology and IRSH. Lumican-deleted *Lum*^–/–^ mice exhibit a loosening of the intertwining of collagen fibers and an increase in interfibrillar space. The difference of dermis size is explained by an increase in interfibrillar space and diameter of type I collagen fibers in the absence of lumican as previously described by Chakravarti and collaborators (Chakravarti et al., 1995, 1998). However, in the present report, spectral analysis of collagen by correlation remains qualitative and requires further investigation to evaluate its properties such as fiber size and orientation (Jeanne et al., 2017) as well as its quantitative contribution and mechanical characteristics (Aziz et al., 2018; Peñuela et al., 2018). It is important to note that IRSH can not only distinguish different structures of the skin but can also specifically target ECM macromolecules such as collagen. From a therapeutic point of view, for example in the context of tumor progression such as melanoma, it will be important to study the drug delivery potential taking into account the role of lumican in ECM integrity. Indeed, an absence of Lumican can potentially increase the intra- and peri-tumoral accessibility of anti-cancer drugs, as described by Jeanne and collaborators (Jeanne et al., 2017).

## Conclusion

We demonstrate here in this study that IRSH represents an interesting approach to identify tissue structures. It is complementary to conventional histology and moreover exhibits some interesting advantages. It avoids the use of different chemicals employed for staining and does not require any labeling. Furthermore, it can be directly applied to paraffin embedded tissues. It allows to visualize the remodeling of the skin tissue in the absence of lumican. Moreover, it can reveal specific histological features in a single analysis without the use of different stainings. It would be interesting to develop a quantitative numerical analysis to evaluate the amount of collagen in the spectral image and compare it with the polarized image with the picrosirius red. Perspectively, it would be interesting to study the impact of lumican in skin tissue by polarized IR spectroscopy, nano-IR spectroscopy and second harmonic generation (SHG) to gain more insight into the organization of collagen fibers.

## Data Availability Statement

All datasets generated for this study are included in the article/Supplementary Material.

## Ethics Statement

This study was performed in compliance with “The French Animal Welfare Act” and following “The French Board for Animal Experiments”. Experiments were conducted under approval of the French “Ministère de l’Enseignement Supérieur et de la Recherche” (ethics committee n°C2EA-56) in compliance with the “Directive 2010/63/UE”.

## Author Contributions

SB and GS contributed to the study conception and design. LN, VU, IP, and SB performed experiments. LN, VU, CB-R, CC-P, SB, and GS contributed to data analysis and interpretation. All authors contributed to the manuscript writing and revision.

## Conflict of Interest

The authors declare that the research was conducted in the absence of any commercial or financial relationships that could be construed as a potential conflict of interest.
