# Supplementary figures and images for: Label-Free Infrared Spectral Histology of Skin Tissue Part I: Impact of Lumican on Extracellular Matrix Integrity
Source: Front Cell Dev Biol. 2020 May 12;8:320. doi: 10.3389/fcell.2020.00320 (PMC7235349; doi:10.3389/fcell.2020.00320)

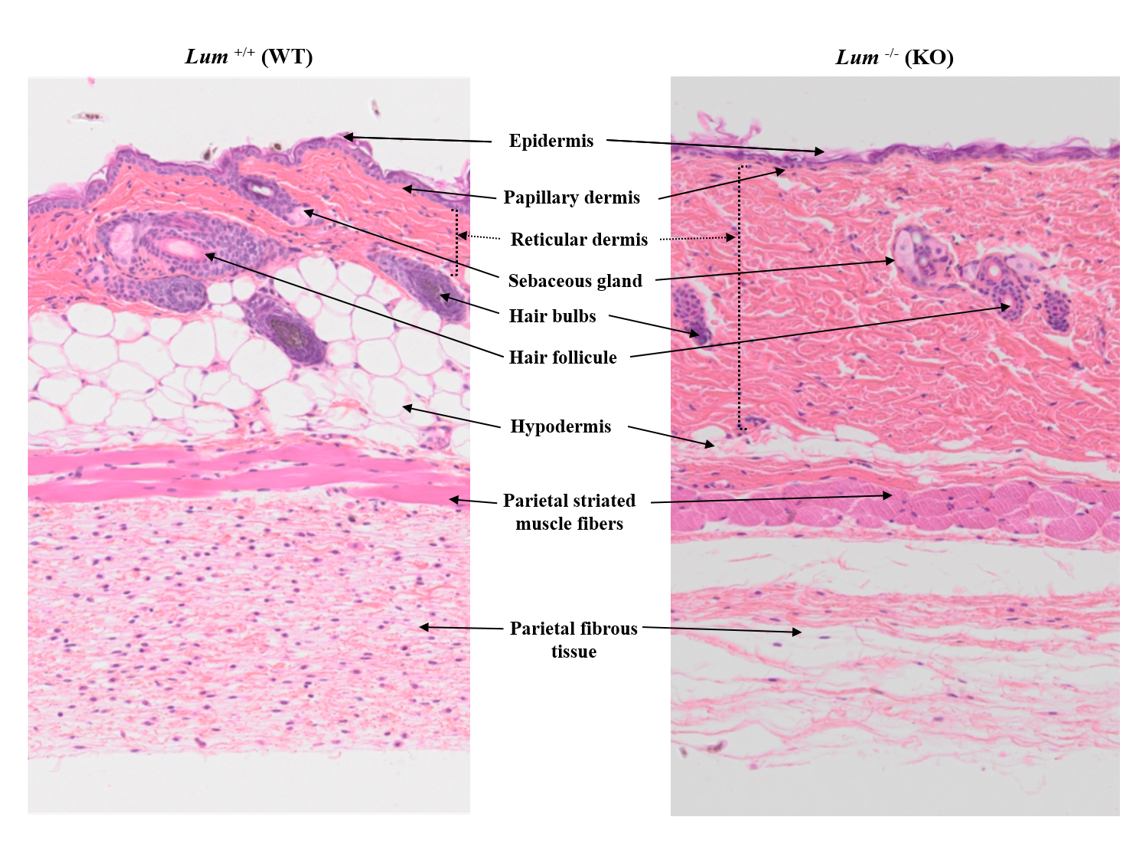

Supplement: Supplementary file 1 [file Image_1.tif]

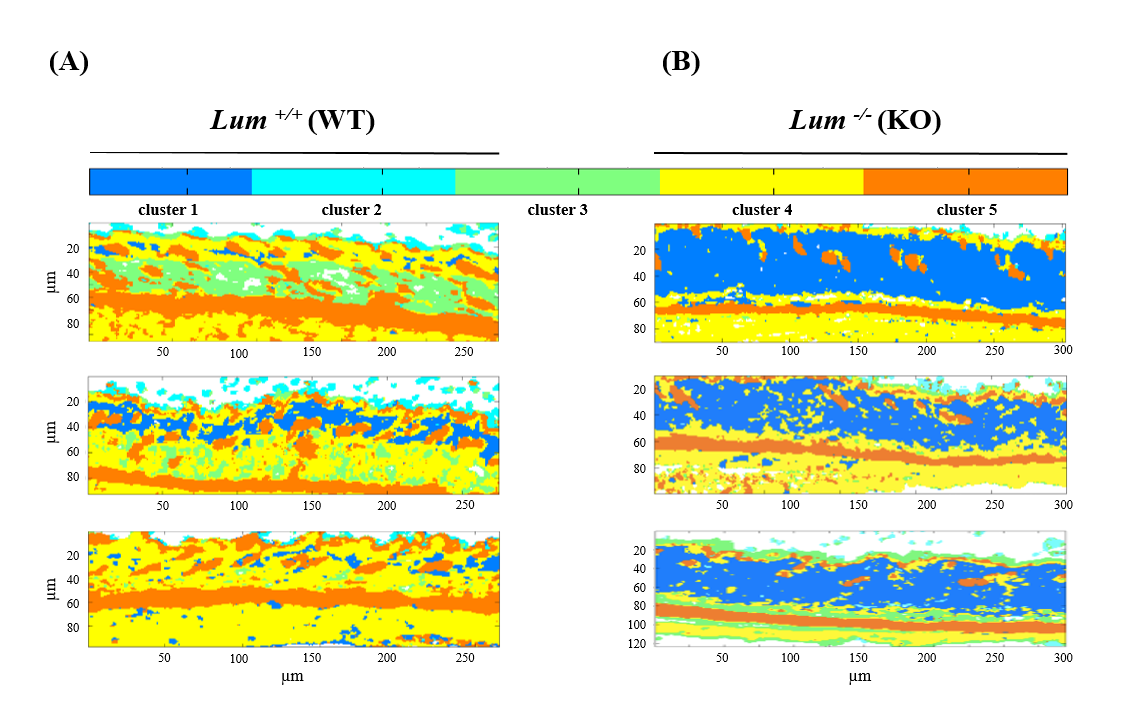

Supplement: Supplementary file 2 [file Image_2.tif]
