# Supplementary material for: Label-Free Infrared Spectral Histology of Skin Tissue Part I: Impact of Lumican on Extracellular Matrix Integrity
Source: Front Cell Dev Biol. 2020 May 12;8:320. doi: 10.3389/fcell.2020.00320 (PMC7235349; doi:10.3389/fcell.2020.00320)
Supplement: Supplementary file 3 [file Table_1.docx]

**Supplementary Table 1 |** Mean percentage contribution of clusters 1-10 after *K*-means clustering of skin tissue FTIR images (n=3).

|  |  | **% contribution of clusters after *K*-means with 10 classes**  *mean ± SEM* | | | | |
| --- | --- | --- | --- | --- | --- | --- |
|  |  |  |  |  |  |  |
| **Cluster number** | | **Cluster 1** | **Cluster 2** | **Cluster 3** | **Cluster 4** | **Cluster 5** |
| ***Lum^+/+^* (WT)** | | 0.61 ± 0.31 | 20.12 ± 2.45 | 1.28 ±0.67 | 5.38 ± 5.28 | 29.73 ± 4.27 |
| ***Lum^-/-^* (KO)** | | 3.11 ± 3.10 | 5.46 ± 1.22 | 9.03 ± 2.48 | 8.60 ± 4.43 | 18.17 ± 4.70 |
| **Cluster number** | | **Cluster 6** | **Cluster 7** | **Cluster 8** | **Cluster 9** | **Cluster 10** |
| ***Lum^+/+^* (WT)** | | 12.02 ± 4.98 | 3.24 ± 1.54 | 6.35 ± 5.20 | 6.33 ± 3.00 | 14.94 ± 1.06 |
| ***Lum^-/-^* (KO)** | | 9.53 ± 2.16 | 1.12 ± 0.26 | 0.65 ± 0.28 | 33.67 ± 3.71 | 10.67 ± 0.52 |
