# Supplementary material for: Label-Free Infrared Spectral Histology of Skin Tissue Part I: Impact of Lumican on Extracellular Matrix Integrity
Source: Front Cell Dev Biol. 2020 May 12;8:320. doi: 10.3389/fcell.2020.00320 (PMC7235349; doi:10.3389/fcell.2020.00320)
Supplement: Supplementary file 4 [file Table_2.docx]

**Supplementary Table 2 |** Mean percentage contribution of clusters 1-5 after *K*-means clustering of skin tissue FTIR images (n=3).

|  |  | **% contribution of clusters after *K*-means with 5 classes**  *mean ± SEM* | | | | |
| --- | --- | --- | --- | --- | --- | --- |
|  |  |  |  |  |  |  |
| **Cluster number** | | **Cluster 1** | **Cluster 2** | **Cluster 3** | **Cluster 4** | **Cluster 5** |
| ***Lum^+/+^* (WT)** | | 7.77 ± 3.69 | 4.30 ± 1.62 | 12.26 ± 6.22 | 45.54 ± 8.35 | 30.13 ± 3.26 |
| ***Lum^-/-^* (KO)** | | 43.31 ± 4.57 | 1.67 ± 0.50 | 7.18 ± 5.42 | 32.47 ± 5.85 | 15.37 ± 1.27 |
